# Endovascular treatment of in-stent restenosis after vertebral artery ostial stenting: incidence, risks and outcomes

**DOI:** 10.3389/fneur.2026.1790256

**Published:** 2026-04-08

**Authors:** Kamran Hajiyev, Sebastian Johannes Müller, Ali Khanafer, Philipp von Gottberg, Uta Hanning, Ketevan Mikeladze, Hans Henkes

**Affiliations:** 1 Neuroradiologische Klinik, Klinikum Stuttgart, Stuttgart, Germany; 2 Klinik für Diagnostische und Interventionelle Neuroradiologie, Medizinische Fakultät Mannheim, Ruprecht-Karls-Universität Heidelberg, Mannheim, Germany; 3 Neuroradiologische Klinik, Universitätsklinikum Magdeburg, Magdeburg, Germany; 4 Medizinische Fakultät, University Duisburg-Essen, Duisburg, Germany

**Keywords:** drug-eluting balloon, drug-eluting stent, endovascular treatment, stenting, vertebral artery ostial stenosis

## Abstract

**Introduction:**

In-stent restenosis (ISRS) is a major concern following vertebral artery (VA) ostial stenting, potentially leading to recurrent symptoms or ischemic strokes. Despite the growing use of endovascular treatment for VA ostial stenosis (VAOS), data on efficacy and safety of the endovascular re-treatment for ISRS is sparse.

**Aim of study:**

To evaluate the incidence of ISRS after VA ostial stenting and assess the safety and effectiveness of endovascular re-interventions.

**Methods:**

This single-center retrospective analysis included patients who underwent re-treatment for ISRS >50% using either balloon angioplasty with a drug-coated balloon (DCB), or re-stenting with drug-eluting balloon-mounted stents (DES). The angiographic follow-up was performed in all cases following re-treatment. Periprocedural neurological events, need for re-treatment, and follow-up outcomes were systematically evaluated.

**Results:**

Over a mean follow-up period of 56 months (range: 9–183 months) after 564 stenting procedures for VAOS in 525 patients, ISRS >50% was found in 89 stents (15.8%), with the majority (70 out of 89) diagnosed within the first year, at a median time of 7 months (range: 1–147 months). Re-treatment was performed in 88 cases, with DES used in 43.2% and DCB in 56.8%. No periprocedural strokes were observed. Recurrent ISRS was detected in 19 stents (21.6%) at a mean of 23.2 months (range: 4–117 months) following re-treatment. Tobacco use and dyslipidemia were significantly associated with the development of ISRS and its recurrence.

**Conclusion:**

The observed ISRS rate underscores the importance of rigorous follow-up after VA ostial stenting. Endovascular re-treatment of high-grade ISRS with DES or DCB appears to be safe and effective, while risk factor management, including smoking cessation, may further reduce recurrence rates.

## Introduction

Vertebral artery ostial stenosis (VAOS) refers to a narrowing at the proximal segment of the vertebral artery (VA), typically at its common origin from the subclavian artery. This condition is mostly caused by atherosclerotic disease and is associated with a significant risk of posterior circulation ischemia, including transient ischemic attacks and vertebrobasilar strokes (1).

VAOS accounts for approximately 20% of cases of posterior circulation ischemia and is often underdiagnosed due to the nonspecific and variable nature of its clinical presentation (2). It can be observed in up to 12.5% of patients over the age of 70 (3) and is frequently associated with other cervical or intracranial stenosis (4).

While medical therapy, including antiplatelet agents and risk factor modification, remains the cornerstone of treatment for most patients, interventional strategies are considered for symptomatic individuals who do not respond adequately to conservative management (5). Endovascular stenting of the vertebral artery origin has emerged as a viable therapeutic option, particularly in patients with recurrent symptoms despite optimal medical therapy or in those with hemodynamically significant stenosis (6–9).

Endovascular treatment involves percutaneous transluminal angioplasty (PTA) followed by stent placement to restore luminal patency and ensure durable revascularization (10). Technological advances in stent design, including drug-eluting stents, have improved procedural success rates and reduced the incidence of restenosis (11).

Despite these advances, vertebral artery origin stenting remains a subject of ongoing investigation. Long-term data regarding in-stent restenosis (ISRS) rates, stroke prevention efficacy, and patient selection criteria are still being developed. Current guidelines recommend individualized decision-making based on symptom burden, lesion severity, and overall vascular anatomy (12). For example, the 2023 ESVS (12) guidelines provide the following recommendation (no. 128): “In patients presenting with a vertebrobasilar territory transient ischemic attack or stroke and a 50–99% vertebral artery stenosis, routine stenting is not recommended.” These recommendations are primarily based on a study by Markus et al. (13), which also suggested that extracranial stenting may be beneficial; however, the authors emphasized that larger, adequately powered clinical trials with long-term follow-up data are necessary to confirm these findings.

The present study builds upon our previously reported experience with vertebral artery ostial stenting in a large single-center cohort (14), which focused primarily on procedural safety and long-term outcomes of the primary intervention. In contrast, the current analysis specifically investigates the incidence, predictors, and endovascular management of ISRS during long-term follow-up, with particular emphasis on treatment strategies and outcomes after re-intervention.

## Materials and methods

This single-center retrospective analysis was conducted in accordance with the Declaration of Helsinki and was approved by the local ethics committee. All patients were informed in detail about the specific use and off-label indications for drug-coated balloons (DCB) and drug-eluting balloon-mounted stents (DES) in VA ostial stenting and re-treatment for ISRS, and written informed consent was obtained from all patients before the procedure.

### Patient selection and evaluation

Patients with VAOS who underwent endovascular stent angioplasty between the 01/01/2008 and 31/12/2022 were retrospectively analyzed. Patients with suspected ISRS > 50%, concordant on Doppler and CT-/MR-angiography imaging, after confirmation by digital subtraction angiography (DSA), were treated with DCB or additional DES implantation. The appropriate treatment method was carefully considered by our neurovascular team based on the morphology of the ISRS, the structural integrity of the stent, as well as the patient’s clinical symptoms and relevant hemodynamic considerations.

The pre-procedural evaluation included assessment of neurological status, stenosis severity, and platelet function testing. ISRS was considered symptomatic if the patient had acute ischemic stroke in the vertebrobasilar territory, vertebrobasilar transient ischemic attack (TIA), imaging evidence of post-ischemic vertebrobasilar lesions, or recurrent vertigo. In patients presenting with recurrent vertigo, alternative etiologies were systematically excluded through neurological, cardiological, and otorhinolaryngological evaluation before attributing symptoms to vertebrobasilar insufficiency. Baseline demographics, risk factors, clinical and periprocedural data were retrospectively collected from our hospital’s database.

### Intervention protocol and technical data

**Figure 1:**
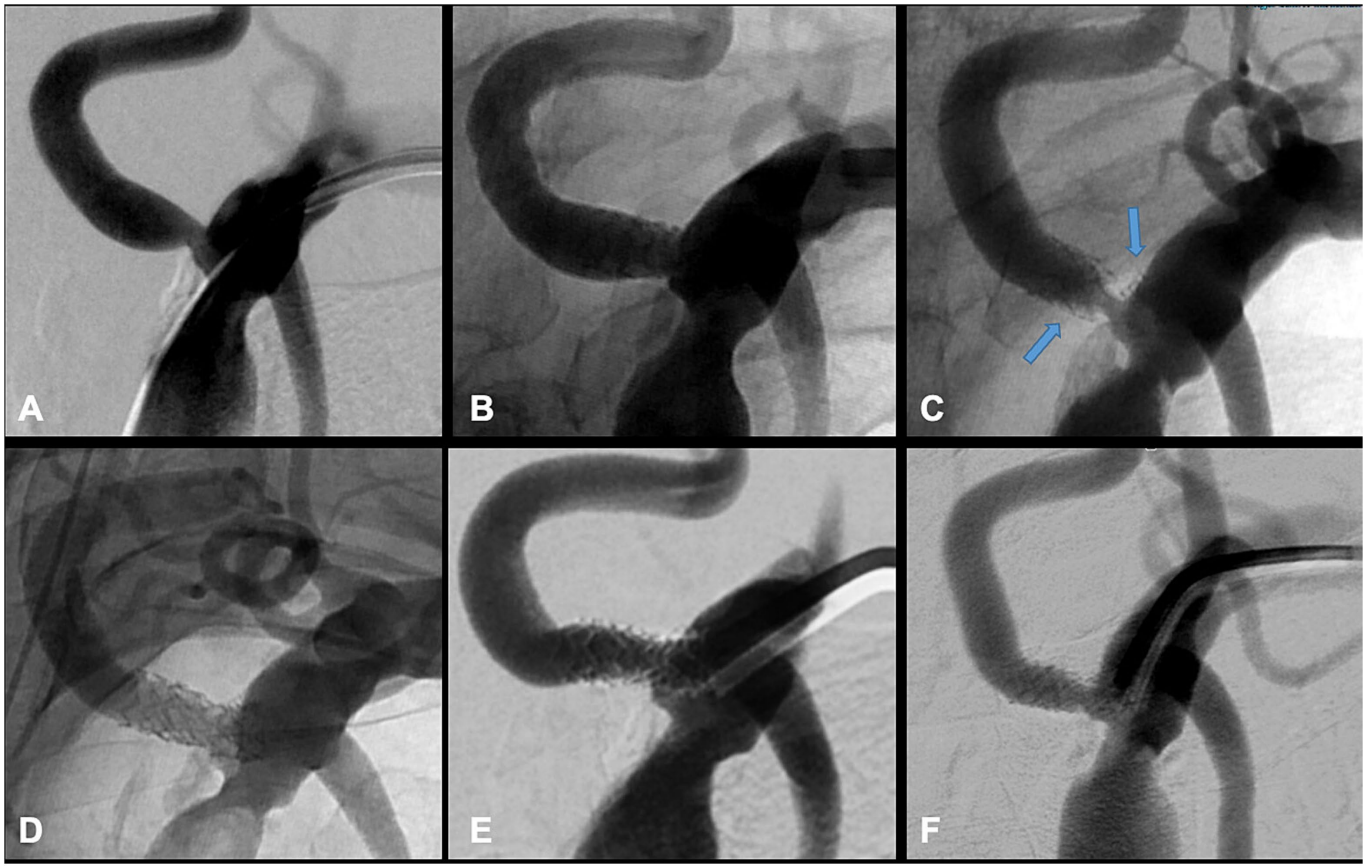
Transradial stenting of a high-grade, symptomatic ostial stenosis of the left VA in the setting of chronic occlusion of the contralateral VA **(A)**. Precise deployment of a balloon-mounted DES at the level of the stenosis is shown **(B)**. Follow-up angiogram after 6 months reveals stent fracture (blue arrows) and high-grade restenosis **(C)**. Re-stenting with an additional DES was performed **(D)**. Follow-up angiograms demonstrate sustained stent patency at 1 year **(E)** and 3 years **(F)**.

Dual antiplatelet therapy with aspirin plus clopidogrel or ticagrelor was initiated 3 days before the procedure. Heart rate and arterial blood pressure were continuously monitored during the procedure. Selective catheterization of the subclavian artery was typically performed with a 6 F guiding catheter. Diagnostic angiography was performed to confirm the precise degree of the ISRS, to exclude the stent fracture and identify a suitable projection. Unfractionated heparin was infused as a 3,000 IU bolus. Embolic protection devices (EPD) were not routinely used due to several factors, including the need for exchange maneuvers, the potential risk of EPD entrapment within the stent, and the lack of supporting evidence for routine EPD use in vertebral artery interventions. A 0.014-in. microguidewire was inserted into the distal V2 segment. A DCB or DEB of the proper size was used to cover the whole length of the ISRS. The DCB was kept inflated for 60–90 s, then deflated and withdrawn. A final angiogram was obtained in all cases. Technical success was defined as restoration of blood flow within the stent, with residual stenosis below 30%. A vascular closure device was preferred for the final repair of the femoral access site. Figure 1 demonstrates examples of a treated VAOS, a re-stenosis with stent fracture, and re-treatment with DES.

### Post-procedural period

After the procedure, all patients underwent in-hospital neurological assessment, and most patients underwent post-procedural brain imaging with MRI before hospital discharge to rule out embolic lesions. Patients were discharged on dual antiplatelet therapy including lifelong aspirin (100 mg/day PO), plus clopidogrel (1 × 75 mg/day PO) or ticagrelor (2 × 90 mg/day PO) for 12 months. Our standard protocol included DSA examinations of the stent in first year after the procedure, and if necessary, during the subsequent follow-up period.

### Statistical analysis

Categorical data are reported as numbers and percentages. The median, mean, minimum, and maximum are reported for continuous variables. Fisher’s exact test was used to determine independence between two categorical variables. Two-sample t-test was used to test the equality of the means of two independent groups. All statistical tests were two-tailed, with a significance threshold of 0.05. Odds ratios (ORs) with 95% confidence intervals (CIs) were calculated to quantify associations between variables and outcomes. Statistical analyses were performed in Stata/IC 16.1 for Unix (StataCorp 4905 Lakeway Drive, College Station, TX 77845, USA). The significance level p was set to 5%.

## Results

### Basic data

A total of 564 stenting procedures were performed in 525 patients (median age ± standard deviation: 70 ± 9.4 years; 146 females) at our single neurovascular center between 2008 and 2022. Thirty-nine patients underwent treatment for VAOS on both sides. Drug-eluting balloon-expandable stents were used in all cases. No significant (>50%) residual stenosis after primary stenting was diagnosed.

**Figure 2:**
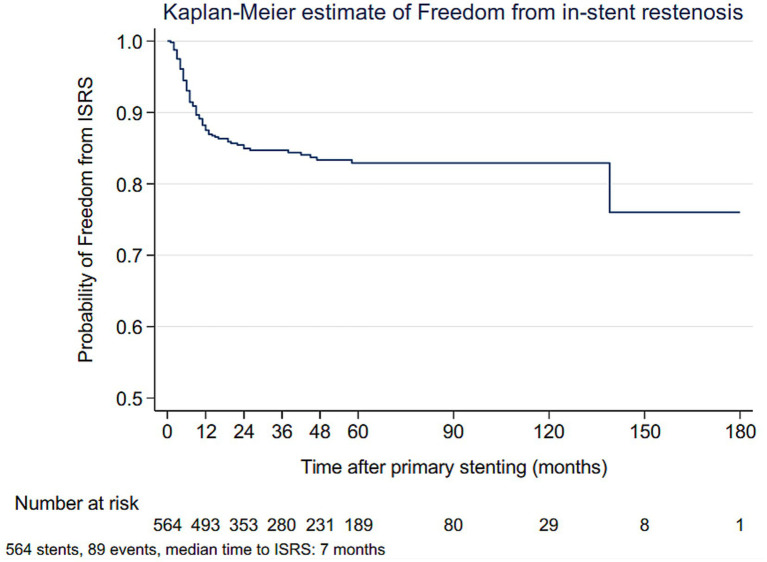
Kaplan–Meier analysis of freedom from in-stent restenosis after vertebral artery ostial stenting. Most restenosis events occurred within the first year after the index procedure, with a median time to ISRS of 7 months.

During an overall mean follow-up of 56 months (range, 9–183 months), ISRS >50% was detected in 89 stents (15.8%), with the majority (70 out of 89) diagnosed within the first year, at a median detection time of 7 months (range, 1–147 months) after the index procedure (Figure 2).

Stent fractures were observed in 26 stents (4.6%), of which half (*n* = 13) were associated with ISRS, accounting for 14.6% of all ISRS cases. One patient experienced a TIA, and six reported recurrent vertigo; no recurrent strokes occurred.

The most common comorbidities in the ISRS group were arterial hypertension (17%), dyslipidemia (20.4%), coronary artery disease (CAD) (19.8%), diabetes mellitus (DM) (16.7%), peripheral artery disease (PAD) (20.9%).

Patient data is summarized in Table 1.

**Table 1:** Patient classification according to baseline demographics and risk factors.

		Interventions (*n*)	In-stent restenosis		Odds ratio (95%-confidence interval)	*p* ^*^
			No	Yes		
All interventions		564	475 (84.2%)	89 (15.8%)		
Group	Asymptomatic	277	236 (85.2%)	41 (14.8%)		
	Symptomatic	287	239 (83.3%)	48 (16.7%)	1.16 (0.73–1.82)	0.565
Gender	Female	156	130 (83.3%)	26 (16.7%)		
	Male	408	345 (84.6%)	63 (15.4%)	0.91 (0.55–1.51)	0.701
Age at treatment (years)	Min.–Max.		41–90	50–83		
	Median		70	69	0.99 (0.97–1.02)	0.415
Atrial fibrillation	No	502	426 (84.9%)	76 (15.1%)		
	Yes	62	49 (79.0%)	13 (21.0%)	1.49 (0.77–2.88)	0.266
Diabetes mellitus	No	390	330 (84.6%)	60 (15.4%)		
	Yes	174	145 (83.3%)	29 (16.7%)	1.10 (0.68–1.79)	0.709
Tobacco use	No	452	392 (86.7%)	60 (13.3%)		
	Yes	112	83 (74.1%)	29 (25.9%)	2.28 (1.37–3.79)	0.002
Arterial	No	110	98 (89.1%)	12 (10.9%)		
hypertension	Yes	454	377 (83.0%)	77 (17.0%)	1.67 (0.87–3.19)	0.145
Peripheral artery disease	No	454	388 (85.5%)	66 (14.5%)		
	Yes	110	87 (79.1%)	23 (20.9%)	1.55 (0.91–2.64)	0.109
Coronary artery disease	No	372	321 (86.3%)	51 (13.7%)		
	Yes	192	154 (80.2%)	38 (19.8%)	1.55 (0.98–2.47)	0.068
Dyslipidemia	No	309	272 (88.0%)	37 (12.0%)		
	Yes	255	203 (79.6%)	52 (20.4%)	1.88 (1.19–2.99)	0.007
Pre-stent stenosis degree	50–75%	168	139 (82.7%)	29 (17.3%)		
	>75%	396	336 (84.8%)	60 (15.2%)	0.86 (0.53–1.39)	0.530

* Significance *p* as determined by Fisher’s exact test.

### Stents

In this study, the following coronary stents were used: Catania and Cobra PzF (CeloNova Biosciences, USA), Coroflex Blue, Coroflex ISAR, Coroflex ISAR NEO, and Coroflex Please (B. Braun Melsungen AG, Germany), RX Herculink Elite (Abbott Vascular, USA), Resolute Integrity and Resolute Integrity RX (Medtronic, USA), as well as Taxus Element (Boston Scientific, USA). All stents were utilized in accordance with the manufacturers’ instructions and evaluated under standardized conditions. Table 2 reveals the distribution of stents and the ISRS rate. Among the various stents used over the years, Coroflex ISAR and Coroflex ISAR Neo (B. Braun, Melsungen, Germany) were significantly associated with a lower incidence of ISRS (*p* = 0.003).

**Table 2:** Incidence of ISRS among various stents.

		Count (*n*)	In-stent restenosis		Odds ratio (95%-confidence interval)	*p* ^*^
			No	Yes		
Name of stent	Catania	2	0 (0%)	2 (100%)		
	Cobra PzF	1	1 (100%)	0 (0%)		
	Coroflex blue	3	1 (33.3%)	2 (66.7%)		
	Coroflex ISAR	157	140 (89.2%)	17 (10.8%)		
	Coroflex ISAR NEO	83	75 (90.4%)	8 (9.6%)		
	Coroflex please	303	246 (81.2%)	57 (18.8%)		
	RX herculink elite	1	1 (100%)	0 (0%)		
	Resolute integrity	7	5 (71.4%)	2 (28.6%)		
	Resolute integrity RX	4	4 (100%)	0 (0%)		
	Taxus element	3	2 (66.7%)	1 (33.3%)		
Coroflex ISAR	No	481	400 (83.2%)	81 (16.8%)		
NEO	Yes	83	75 (90.4%)	8 (9.6%)	0.53 (0.24–1.14)	0.105
Coroflex	No	324	260 (80.2%)	64 (19.8%)		
ISAR + ISAR NEO	Yes	240	215 (89.6%)	25 (10.4%)	0.47 (0.29–0.78)	0.003

* Significance *p* as determined by Fisher’s exact test.

### Re-treatment

**Figure 3:**
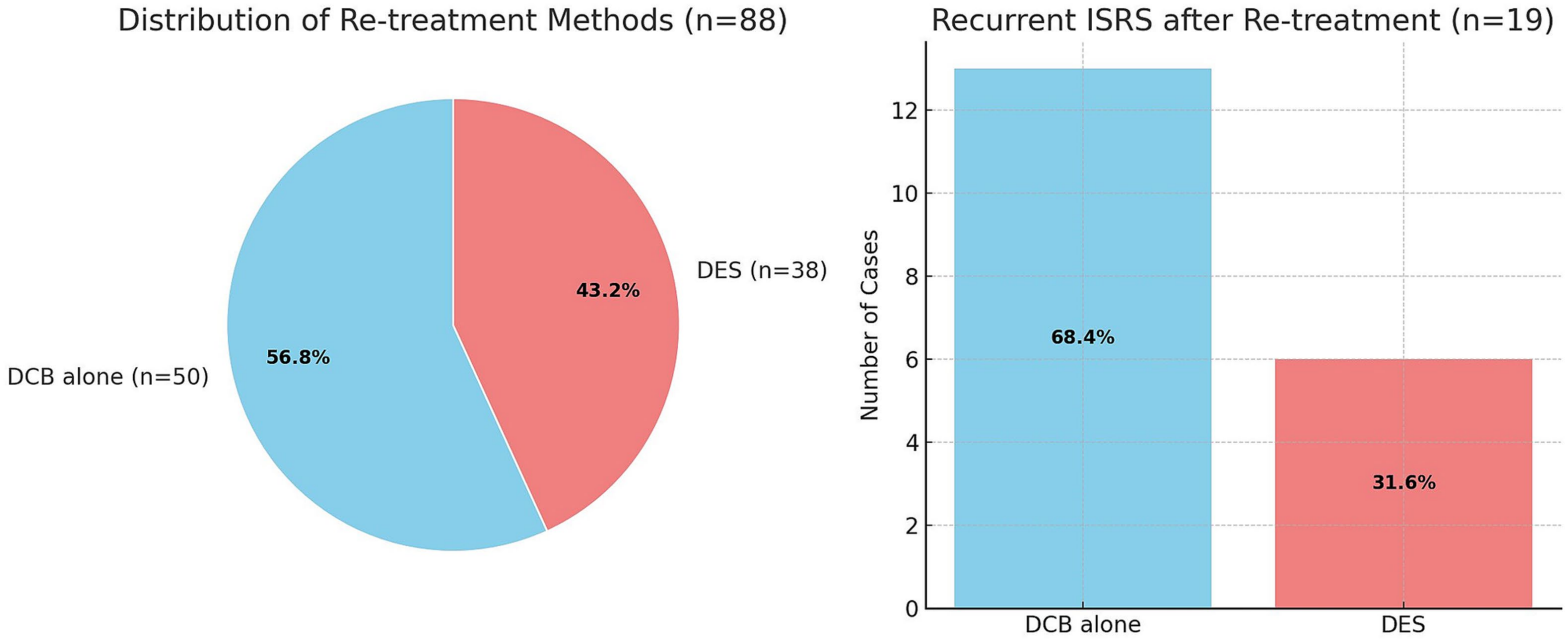
Pie chart (left): Distribution of re-treatment methods with drug-coated balloons (DCB) alone (*n* = 50) and drug-eluting stents (DES, *n* = 38). Bar chart (right): Recurrent ISRS after re-treatment occurred in 19 cases (21.6%), treated either with DCB alone (*n* = 13, 68.4%) or DES (*n* = 6, 31.6%).

One patient declined re-intervention. Re-treatment was performed in 88 cases, using drug-coated balloons (DCB) alone in 50 cases (56.8%). In 38 cases (43.2%), a new stent was implanted—either as primary treatment (*n* = 33) or as an additional stent following DCB angioplasty (*n* = 5; Figure 3). The median intervention time was 23 min, and the median X-ray exposure time was 10 min. No periprocedural complications observed. During further follow-up after re-treatment, asymptomatic recurrent ISRS >50% was observed in 19 stents (21.6%) at a mean of 23.2 months (range, 4–117 months). Of these, 13 cases (68.4%) had been re-treated with DCB and 6 cases (31.6%) with re-DES. A significantly higher incidence of ISRS and recurrent ISRS was observed in patients with dyslipidemia (20.4%, *p* = 0.007) and a history of tobacco use (25.9%, *p* = 0.002).

## Discussion

In our cohort of 525 patients with 564 stents, ISRS >50% occurred in 89 stents, with a median detection time of 7 months (14). Re-treatment was successfully performed in 88 stents, with no periprocedural complications or recurrent strokes documented during long-term follow-up of up to 15 years. These findings indicate that both primary stenting for VAOS and endovascular re-intervention for ISRS can be performed safely in high-volume centers with appropriate expertise. Numerous studies confirm the safety and feasibility of vertebral artery ostial stenting (15–18). For example, Lin et al. (19) and Ogilvy et al. (20) reported favorable acute outcomes and acceptable long-term patency using coronary stents. Similarly, El Koussa et al. (21) demonstrated low periprocedural complication rates in a contemporary cohort.

Importantly, long-term outcome data from Karameshev et al. (22) and Tayler et al. (23) emphasize sustained symptom relief and low rates of recurrent stroke in high-volume centers.

Restenosis remains a challenge, with incidences reported between 10 and 40% depending on follow-up duration and stent type (24, 25). With a restenosis rate of 15.8%, our results were within the range reported in the literature, but at the lower end of the spectrum.

Stent type emerged as an important predictor of ISRS. A randomized multicenter trial by Si et al. (26) reported that DES implantation in symptomatic intracranial and vertebral artery stenosis led to a significantly reduced 180-day ISRS rate (14.5% with DES vs. 43.8% with BMS, absolute reduction 29.3%). The trial further demonstrated no differences in technical success or periprocedural adverse events between DES and BMS, supporting the comparable safety profile of both stent types. These findings align with our rationale for selecting DES as the preferred device in our series. Furthermore, advancements in stent technology have improved outcomes. Ortega-Gutierrez et al. (11) demonstrated that second-generation DES were associated with lower restenosis rates. In our larger cohort, we likewise observed favorable results with newer-generation DES.

Taylor et al. (27) revealed that cigarette smoking was associated with increased binary restenosis rates. Zheng et al. (28) identified residual stenosis, stent diameter, and alcohol consumption as key risk factors. Our analysis also identified nicotine consumption and dyslipidemia as risk factors, with the latter representing a novel observation in comparison to existing literature. Lifestyle modification and management of comorbidities may therefore influence the risk of ISRS.

Stent fractures, well-documented in coronary interventions, are also not uncommon after vertebral artery ostial stenting (29). Contributing factors include pulsatile motion, vessel angulation, and the anatomical bending point at the VA origin. Reported fracture rates range from 5 to 15% within the first year and may increase to up to 30% over long-term follow-up, and can possibly lead to higher incidence of ISRS (30). In our cohort, stent fractures were identified in 4.6% of cases. Nearly half of these fractures were associated with the development of significant ISRS, representing 14.6% of all restenosis cases observed during follow-up.

The incidence of ISRS can be further influenced by hemodynamic factors, including the technique of stent deployment during primary treatment. Qiao et al. (31) used computational fluid dynamics (CFD) modeling to demonstrate that stent-induced geometric changes can cause flow disturbances, and identified an optimal stent protrusion of 1 mm into the subclavian artery to minimize these effects. Various techniques have since been introduced to optimize precise stent placement at the ostium, aiming to reduce geographic miss and improve long-term patency (32, 33).

To address in-stent restenosis, DCB angioplasty has emerged as a promising option. A recent single-center experience by Xu et al. (34) showed encouraging patency rates after DCB for ISRS after stenting, with no major adverse events reported. Re-stenting with a stent-in-stent approach may represent a reasonable option, particularly in cases of stent fracture.

Given the evolving landscape of VAOS treatment, the decision to perform revascularization, especially in cases of restenosis or stent fracture, must be individualized. As demonstrated in our study and previous reports, such procedures can be performed with acceptable safety in experienced, high-volume centers. The integration of DES, DCB, precise deployment techniques, and careful patient selection may contribute to improved outcomes.

### Limitations

This study has several limitations. First, the retrospective single-center design may limit generalizability. Second, follow-up duration varied widely among patients, and imaging surveillance was performed according to clinical practice rather than at strictly predefined intervals. Consequently, the timing of restenosis detection may be influenced by differences in follow-up schedules.

Third, the analysis was performed on a per-stent basis, as restenosis represents a device-related outcome. However, some patients underwent bilateral vertebral artery stenting, which may introduce clustering effects and limit statistical independence between observations.

Finally, the statistical analyses were primarily exploratory and based on univariate comparisons. Because the number of restenosis events relative to candidate predictors was limited, multivariable regression analysis was not performed. Therefore, the associations identified for tobacco use and dyslipidemia should be interpreted as hypothesis-generating findings rather than causal relationships.

### Outlook

In this single-center analysis with a mean follow-up of 5 years (up to 15 years in some patients), we were able to provide a robust median assessment. Nevertheless, future prospective, multicenter studies are needed to validate these findings and to better understand the impact of individual clinical decisions on patient outcomes. Standardized treatment protocols may help to reduce variability and improve comparability across centers.

## Conclusion

The observed ISRS rate underscores the importance of rigorous follow-up after VAOS to enable early detection and timely management. Our findings suggest that endovascular re-treatment of high-grade ISRS using DES or DCB appears feasible and safe in experienced centers. In addition, addressing vascular comorbidities and promoting lifestyle modifications may further reduce the risk of ISRS and improve long-term outcomes.

## Data Availability

The original contributions presented in the study are included in the article/supplementary material, further inquiries can be directed to the corresponding author.
